# An Account of the Practice of One of the Physicians of the Westminster General Dispensary, and of the Western Dispensary; from March 20 to April 20, 1806

**Published:** 1806-05

**Authors:** Samuel Fothergill

**Affiliations:** Southampton Street, Strand


### *An Account of the Practice of one of the Physicians of the Westminster General Dispensary, and of the Western Dispensary; from March 20 to April 20, 1806.*

| | |
|---|---|
| **Acute Diseases.** | |
| Continued Fever | 2 |
| Scarlet Fever and Sore Throat | 3 |
| Measles | 1 |
| Peripneumony | 1 |
| Hæmoptoe | 1 |
| Catarrh | 10 |
| Acute Rheumatism | 4 |
| Colic | 2 |
| Dysentery | 1 |
| **Chronic Diseases.** | |
| Cough and Dyspnœa | 38 |
| Pulmonary Consumption | 2 |
| Scrophula | 2 |
| Rheumatism | 5 |
| Lumbago | 6 |
| Cephalalgia and Vertigo | 5 |
| Gastrodynia | 6 |
| Enterodynia | 3 |
| Diarrhœa | 5 |
| Asthenia | 8 |
| Dropsy | 2 |
| Pyrosis | 1 |
| Jaundice | 3 |
| Vomiting | 2 |
| Abscess of the Liver | 1 |
| Dysphagia | 1 |
| Dysuria | 1 |
| Deafness | 1 |
| Psora | 5 |
| Syphilitic Eruption | 2 |
| **Periodical Diseases.** | |
| Quotidian | 3 |
| Hemicranium | 1 |
| Tic Douloureux | 1 |

Dis-

Diseases of Women and Children.

Chlorosis & Amenorrhœa 4

Menorrhagia - - - - 3

Puerperal Fever - - - - 1

Inflammation of the Breast 1

Abortus - - - - - - 1

Gravid Uterus with Pyrexia 2

Prolapsus Uteri - - - 1

Leucorrhœa - - - - - 3

Hysteria - - - - - - 2

Hooping Cough - - - - 1

Scarlatina - - - - - - 2

Small-pox - - - - - - 1

Pyrexia from teething - 1

Peripneumony - - - 1

Catarrh - - - - - - 3

Scrophula - - - - - - 1

Worms - - - - - - - 2

Diarrhœa - - - - - - 2

Scald Head - - - - - 1

Since the last Report, catarrhal fever, cough and dyspnœa, have been very general throughout the metropolis and its vicinage. In some instances the symptoms were obstinate, and many of the patients were affected with pain in the chest and side, which only yielded to blistering. Acute rheumatism has also been a frequent and troublesome complaint; the difficulty of cure has, in some cases, been augmented by the accession of cough and affections of the chest. In this town, bleeding is now seldom used in the cure of acute rheumatism; for though present relief be obtained by the use of the lancet, the symptoms generally return, and the patient is less able to combat with their violence; if, however, blood must be drawn, topical bleeding with leeches offers the most effectual means of reducing the inflammation, without materially affecting the strength of the patient. In Scotland a very common and a very successful mode of practice, is to place the patient between blankets, give frequent doses of pulvis ipecacuanhæ compositus, with copious diluting beverage, and thus keep up a profuse perspiration for several hours, when a complete cure is often effected. This practice is less beneficial in London, where the atmosphere is less pure, and the habits of the people less conducive to health; I have tried it in several cases, while in others I have used bark, sometimes with antimonials, and sometimes with acids, occasionally giving opium; the result of about twenty cases, is in favour of the latter mode of practice. Sleep is often procured, a remission of all the symptoms obtained, and the cure materially hastened by the judicious exhibition of opium.

During the greatest part of the month, the wind was easterly; and with the exception of three or four fine days, the weather has been cold, and some snow has fallen. To this severe commencement of spring, after a wet and mild winter, may safely be attributed the cause of the numerous pulmonic and catarrhal affections, noticed in this month's report.

Two of the cases of sore throat were attended with fever and scarlet eruption; one of them occurred in a private patient. She had for a month previous to the attack, laboured under great anxiety of mind and depression of spirits, had confined herself to the house, and almost to one apartment, when she began to complain of sore throat and fever. On the third day the fever was disposed to be malignant; the skin was dry, the heat very little increased beyond the natural standard, the tongue furred, with a quick feeble pulse, and great prostration of strength. She complained of much pain in her head and bones, great difficulty of swallowing, and was very faint and unwilling to use the least exertion; in consequence of which, the throat was not inspected till the following day, when a deep ulcer was observed on the right tonsil. Large scarlet blotches now appeared on the arms and about the breasts; and though not delirious, she was extremely low, and averse from taking nourishment or medicine. The consequence of such conduct being insisted upon, she consented to take the medicines. A blister had been applied round the throat, and bark was now given in decoction and compound tincture, as freely as the stomach would bear; and the bowels were gently opened with calomel and rhubarb. On the eighth day, she began to mend, desquamation of the cuticle took place, and she soon had only to contend with debility. The complaint was not traced to contagion; no person in the family was affected by it, and she had not previously been out of doors for several weeks. One of the other cases, was in a child aged ten years, and differed from the above, in being attended by delirium, and the scarlet eruption was more generally diffused over the surface of the skin. The treatment was nearly the same, and the termination equally favourable. In both cases, a small dose of calomel and rhubarb given at night, was evidently beneficial, the tongue, in the morning, being much cleaner, and the patient more cheerful and alert.

SAMUEL FOTHERGILL.

*Southampton Street, Strand,*
*April* 22, 1806.

MEDICAL

